# Quantifying the Evolution of Vascular Barrier Disruption in Advanced Atherosclerosis with Semipermeant Nanoparticle Contrast Agents

**DOI:** 10.1371/journal.pone.0026385

**Published:** 2011-10-18

**Authors:** Huiying Zhang, Lei Zhang, Jacob Myerson, Kristin Bibee, Michael Scott, John Allen, Gregorio Sicard, Gregory Lanza, Samuel Wickline

**Affiliations:** 1 Department of Medicine, Washington University, St Louis, Missouri, United States of America; 2 Department of Biomedical Engineering, Washington University, St Louis, Missouri, United States of America; 3 Department of Cellular Biology, Washington University, St Louis, Missouri, United States of America; 4 Department of Surgery, Washington University, St Louis, Missouri, United States of America; University of Frankfurt - University Hospital Frankfurt, Germany

## Abstract

**Rationale:**

Acute atherothrombotic occlusion in heart attack and stroke implies disruption of the vascular endothelial barrier that exposes a highly procoagulant intimal milieu. However, the evolution, severity, and pathophysiological consequences of vascular barrier damage in atherosclerotic plaque remain unknown, in part because quantifiable methods and experimental models are lacking for its *in vivo* assessment.

**Objective:**

To develop quantitative nondestructive methodologies and models for detecting vascular barrier disruption in advanced plaques.

**Methods and Results:**

Sustained hypercholesterolemia in New Zealand White (NZW) rabbits for >7–14 months engendered endothelial barrier disruption that was evident from massive and rapid passive penetration and intimal trapping of perfluorocarbon-core nanoparticles (PFC-NP: ∼250 nm diameter) after *in vivo* circulation for as little as 1 hour. Only older plaques (>7 mo), but not younger plaques (<3 mo) demonstrated the marked enhancement of endothelial permeability to these particles. Electron microscopy revealed a complex of subintimal spongiform channels associated with endothelial apoptosis, superficial erosions, and surface-penetrating cholesterol crystals. Fluorine (^19^F) magnetic resonance imaging and spectroscopy (MRI/MRS) enabled absolute quantification (in nanoMolar) of the passive permeation of PFC-NP into the disrupted vascular lesions by sensing the unique spectral signatures from the fluorine core of plaque-bound PFC-NP.

**Conclusions:**

The application of semipermeant nanoparticles reveals the presence of profound barrier disruption in later stage plaques and focuses attention on the disrupted endothelium as a potential contributor to plaque vulnerability. The response to sustained high cholesterol levels yields a progressive deterioration of the vascular barrier that can be quantified with fluorine MRI/MRS of passively permeable nanostructures. The possibility of plaque classification based on the metric of endothelial permeability to nanoparticles is suggested.

## Introduction

The atherothrombotic syndromes of coronary heart disease, ischemic stroke and peripheral artery disease together account for 22% of all deaths worldwide (2004), with a prevalence of 26 million cases in the USA alone commanding a cost of greater than $4 billion/yr (2006) [1]. The cause of acute vascular syndromes is attributable to rupture of the thin cap overlying an inflamed, fatty atherosclerotic plaque in 2/3 of cases, and to endothelial “erosions” in the rest [2], [3], [4], [5]. In either case, the normally restrictive vascular barrier function is disturbed as a consequence of inflammatory cellular infiltrates, reactive oxygen species, and growth factors that result in enhanced local permeability, inflammatory cell transcytosis, and thrombogenicity [6], [7], [8], [9], [10], [11], [12]. In the early stages of the process, enhanced endothelial penetration and retention of lipoproteins has been hypothesized to incite inflammatory events that lead to plaque progression in a process that proceeds by years any clinical presentation [13]. However, the evolution and ultimate severity of endothelial permeability in response to long-term hypercholesterolemia has not been elucidated in experimental models or clinical subjects.

The focal pathophysiological features that mark the evolution of late stage barrier disruption remain uncertain as a consequence of the lack of well accepted and robust experimental models that suitably represent the complexity of endothelial barrier disruption [14], and the lack of specific nondestructive diagnostic techniques that can quantify lesion severity. Acute experimental models of lipotoxic stress have been developed that elicit endothelial cell apoptosis as a precursor to vascular barrier disruption [15], [16]. Increased endothelial permeability is an early preclinical feature of barrier dysfunction and has been studied with the use of tracer agents (<5 nanometers) such as horse radish peroxidase, radiolabeled albumin, or various permeable dyes [7], [17]. Over time, endothelial cell senescence ensues, leading to local inflammatory responses, compromised endothelial regenerative potential, and ultimately apoptosis and endothelial denudation [18], [19], [20], [21], [22]. Yet the aforementioned methods for studying endothelial function do not specifically depict barrier disruption *in vitro* or *in vivo* because they generally reflect paracellular leakage in early stages of disease, as distinguished from later stage endothelial disruptions (erosion, micro-tears, etc.) that might portend more immediate clinical consequences.

To develop a methodology ultimately capable of longitudinally delineating the severity of endothelial barrier disruption, we propose a translatable diagnostic imaging approach based on the use of semipermeant, diffusible PFC-NP [23], [24], [25] tracers that might penetrate highly permeable late stage plaques. The fluorine core of these nanostructures can be sensitively registered with the use of magnetic resonance spectroscopy and imaging to provide a unique diagnostic marker for leaky endothelial barriers. We hypothesized that the larger dimension of these particles (∼250 nanometer, nominal diameter), as compared with smaller circulating lipoproteins known to penetrate and collect in lesions at a very early stage [13], would manifest limited diffusion into minimally diseased regions in early plaques that retain a relatively intact vascular barrier, while enabling registration of more severe endothelial disruption in advanced plaques.

The quest to develop a methodology that predicts plaque behavior has occupied researchers for many years. Unfortunately, new data from the multicenter PROSPECT trial mapping the natural history of vascular events after initial presentation for acute coronary syndromes indicate that single structural predictors such as thin cap atheroma, plaque burden, or luminal stenosis suffer poor specificity for predicting subsequent events over a 3.4 year interval (∼5–10%). Thus, these classic features of plaque behavior, while seemingly necessary to establish the conditions for plaque rupture, are not sufficient to classify risk in individual patients [26]. The image-based readouts proposed herein may enable for the first time a quantitative description of the development of unexpectedly severe endothelial permeability in advanced experimental atherosclerosis and suggest a method for *in vivo* interrogation of heretofore uncharacterized biophysical features of advanced plaques according to a quantifiable functional permeability metric rather than the structural readouts employed in trials such as PROSPECT.

## Results

### Temporal evolution of vascular permeability

**Figure 1:**
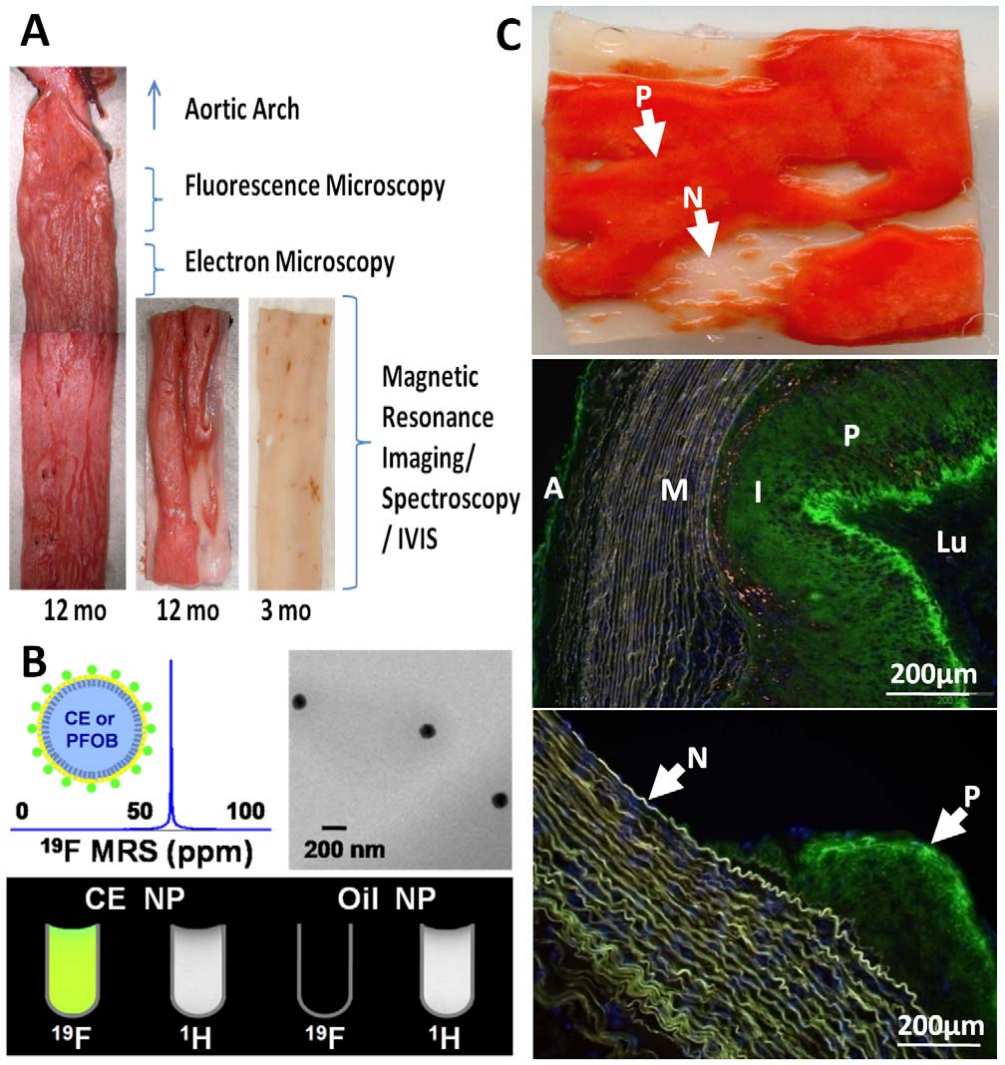
Rabbit aorta cholesterol plaque formation and NP diffusion into intima *in vivo*. A: Sudan IV staining (en face) of opened aorta section showing plaque (red) in 12 month diet rabbit but seldom in 3 month diet rabbit. Sections were collected as labeled for fluorescence microscopy and histology (1 cm), electron microscopy (0.5 cm), lower segments for MRI/MRS and whole mount fluorescence images (IVIS). B: Cartoon of PFC-NP structure, and ^19^F MR spectroscopy (top left), TEM of NP (top right); ^19^F and ^1^H MR image of test tubes containing CE core NP showing fluorine signatures (bottom) and oil core NP as control showing no signal. C: Top: En face Sudan IV staining of section with plaque (P: red) and grossly normal (N: clear) areas. Mid: Marked fluorescent nanoparticle presence in plaque (P) intima (I) of 12 mo cholesterol diet rabbit aorta (green) after 12 hours *in vivo* circulation. Minimal staining of adventitia (A) is noted , and none apparent in media (M) or lumen (Lu). Bottom: Fluorescent NP signals (green) in plaque intima (P), but not the adjacent grossly normal regions (N). Blue = DAPI nuclear stain.

A synopsis of all animal experiments is tabulated in the supplemental data (**Table S1**). Serum cholesterol levels measured at baseline and periodically (every 1–3 months) increased from 1.45±0.13 mg/dl to of an average of 27.30±1.27 mg/dl for all multiple samples for each rabbit. All 27 rabbits fed with high cholesterol diet exhibited atherosclerotic plaques. The three month diet rabbits exhibited minimal plaque protrusion and thinner plaques overall, more prominent at expected branch points, with no apparent vessel remodeling. Rabbits on diet for more than 7 months exhibited more severe atherosclerotic lesions, extending throughout the entire extent of the collected specimen in 12 month diet animals, but regionally patchy in many cases with less affected areas adjacent to severely affected ones (see **Figure 1A and C**). We hypothesized that the semipermeant nanoparticles illustrated in **Figure 1B**, which depicts the properties of the fluorine nanoparticle contrast agent for imaging and spectroscopy based on the unique ^19^F signals that emanate from the Crown Ether (CE) core, would penetrate the raised plaques but not the adjacent more normal appearing regions (**Figure 1C** **top**), or the earlier 3 month old lesions.

For all rabbits beyond the 7 month feeding interval, massive penetration and accumulation of trapped NP were observed in the intimal regions of all plaques so treated, but few in the adventitia and none apparent in the media, indicating *in vivo* penetration from the luminal side. In the aorta samples, a spatially heterogeneous distribution of plaques and particle accumulation was observed, with no particles apparent in adjacent more normal appearing intima in the same tissue sections (**Figure 1C** **center/bottom**). As a further control, we also noted that nonfluorescent nanoparticles produced no intimal optical signal in two samples (**Figure S1A, B**), indicating the autofluorescence was not confounding.

**Figure 2:**
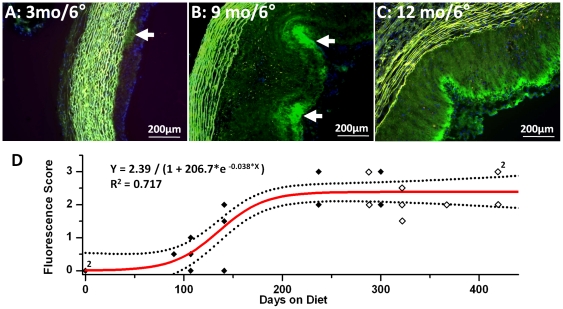
Duration of cholesterol feeding determines NP plaque penetration *in vivo*. A: 6 hours circulation *in vivo* with Alexa fluor-488 labeled NP in 3 month cholesterol diet rabbit aorta showing modest intimal plaque thickening and little fluorescent signal (arrow). Note lack of green-yellow autofluorescence in intima and + nuclear (DAPI stain). B: *In vivo* 6 hours circulation with identical NP in 9 month cholesterol diet rabbit aorta indicative of marked endothelial penetration and particle trapping (arrow). C: *In vivo* 6 hours circulation with identical NP in 12 month cholesterol diet rabbit aorta showing extensive NP penetration into intima (green). D: Sigmoidal fitting of fluorescence scores of aortas exposed *in vivo* to fluorescent NP circulating for 2 hours (open) and 6 hours (solid) on rabbits fed with cholesterol for 0 to 419 days. The temporal dependence of plaque permeability on the duration of hypercholesterolemia is well approximated with a sigmoidal function.

Cholesterol diet duration also influenced *in vivo* nanoparticle penetration as shown by the very limited plaque and intimal signals in rabbits fed cholesterol for only 3 months (**Figure 2A**) versus more extended exposure times (**Figure 2B, C**), suggesting the necessity for prolonged exposure to induce plaque growth and significant endothelial barrier disruption. This very spotty and limited plaque penetration at three months was characteristic of the relatively preserved barrier function even in the presence of slightly raised plaques as evidenced by the DAPI nuclear staining of intimal thickening in **Figure 2A**. Again, grossly unaffected regions did not accumulate nanoparticles at any time point.

A plot of fluorescence scores for all rabbits exposed to 2 (n = 9) or 6 (n = 13) hour NP circulation (**Figure 2D**) illustrates the temporal dependence of plaque permeability on the duration of hypercholesterolemia. Because most experiments allowed *in vivo* nanoparticle circulation for 2 or 6 hours, which manifested statistically equivalent plaque penetration by ANOVA (p = 0.28), the fluorescence scores at these time points were plotted for all rabbits at all feeding times and were fitted nicely to a sigmoidal function (R^2^ = 0.717) to illustrate the temporal progression of NP penetration. An exponential fit also was statistically significant (R^2^ = 0.678). Furthermore, the lack of difference between 2 hour and 6 hour circulation times for fluorescence scores attests to the rapidity of the diffusion process. Limited samples for 1 (n = 1), 12 (n = 1), and 24 (n = 1) hour circulating times indicated similarly rapid nanoparticle trapping (data not shown).

### Quantification of nanoparticle signal in plaque

**Figure 3:**
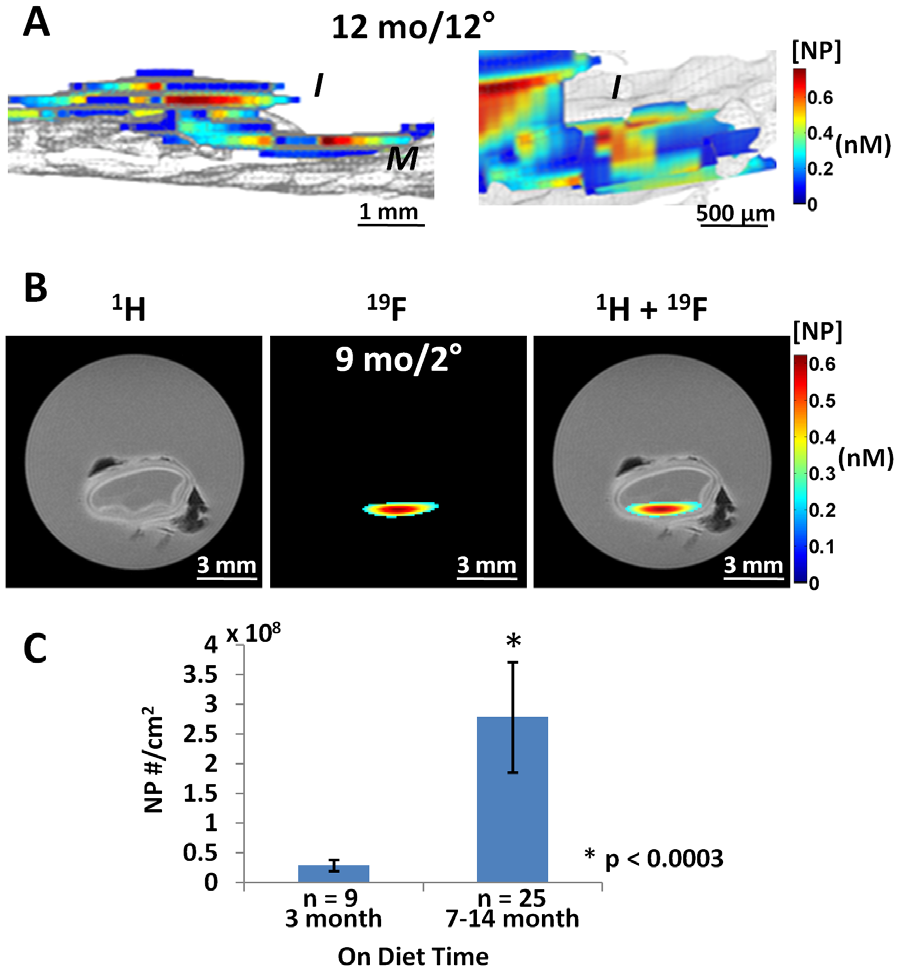
MRI imaging and quantification of NP signal in cholesterol plaques. A: *Left*: 3D saggital rendering of nanoparticle signals from aorta of 12 mo cholesterol fed rabbit after 12 hours circulation time *in vivo*. ^19^F MR image registering nanoparticle fluorine cores (color) overlaid on ^1^H MR image (gray) of aorta show intimal (I) location of particles trapped in thickened plaque (color) atop the medial (M) layer (gray). Particle concentration per voxel is coded in nM (scale bar). *Right*: Close up of intimal layer. B: ^1^H, ^19^F and overlay MR transverse images of aortic rings with nanoparticles trapped in intima of thickened plaque from 9 mo cholesterol fed rabbit after 2 hour circulation *in vivo*. Black artifacts are small air bubbles. Note lack of ^19^F signal from more normal adjacent tissue sections. C: Comparison of normalized CE NP number to the endothelial surface area between 3 month diet and >7 month diet rabbit aorta samples showing 10 fold greater accumulation in older plaques.

To confirm the presence of *intact nanoparticles* in the intima after *in vivo* circulation, coregistered fluorine (^19^F) and proton (^1^H) magnetic resonance images acquired *ex vivo* at 11.7T revealed abundant ^19^F signals emanating from the nanoparticle CE cores in the intima of raised plaques but apparently absent from the adjacent grossly normal regions, consistent with the prior fluorescent images (**Figure 3A, B**). In 25 aortic segments from 16 rabbits with advanced lesions (>7 months feeding) a quantifiable ^19^F MR spectrum was obtained resulting in an average concentration of PFC-NP of 2.78±0.93×10^8^/cm^2^ across the entire tissue slice (**Figure 3C**). On the contrary, in 9 segments from four 3-month diet rabbits with less severe plaques, significantly reduced permeation of PFC-NP was detected, measuring only about 10% of that for the older plaques, or 0.28±0.10×10^8^/cm^2^ (p<0.0003).

For the ^19^F MR imaging, 12 segments from 10 rabbits demonstrated heterogeneously distributed fluorine signals in areas of raised plaque but no detectable fluorine signal in adjacent areas (**Figure S1C, D**) that appeared grossly normal, irrespective of the fluorescence signals (**Figure S1A, B**). These images (**Figure S1A, C**) also indicate that the fluorescent signals originate from intact nanoparticles since the ^19^F signals are present in the same areas where fluorescence is positive, but not in adjacent grossly normal regions (also **Figure 3B**).

Nanoparticle treated specimens could be identified from fluorescence signals in intact whole mount preparations (**Figure S2A**) that permitted further quantitative segmental analysis with high resolution MRI/MRS (**Figure S2B,C**). As we have demonstrated previously [27], [28], [29] the ^19^F CE spectral signatures were collected along with a perfluorooctylbromide (PFOB) reference signal (**Figure S2B**) to permit absolute quantification of the concentration of tissue bound nanoparticles per voxel, shown in **Figure S2C** as *nanomolar* color-coded particle concentrations. In this example the signal-to-noise for CE and PFOB in the region-of-interest shown in **Figure S2B** was 29.7 dB and 26.2 dB respectively; and ^19^F CE signal integration revealed a total volume of PFC-NP of 4.5 nanoliters, or 6.05×10^8^ particles in the sample of 10 month diet rabbit. As shown in **Figure S2C right**, these quantitative measures of tissue PFC-NP content also may be expressed alternatively as *moles* of either PFC-NP or ^19^F atoms, or as *molar* concentrations of either moiety.

### *Ex vivo* confirmation of nanoparticle penetration through disrupted endothelial barriers

**Figure 4:**
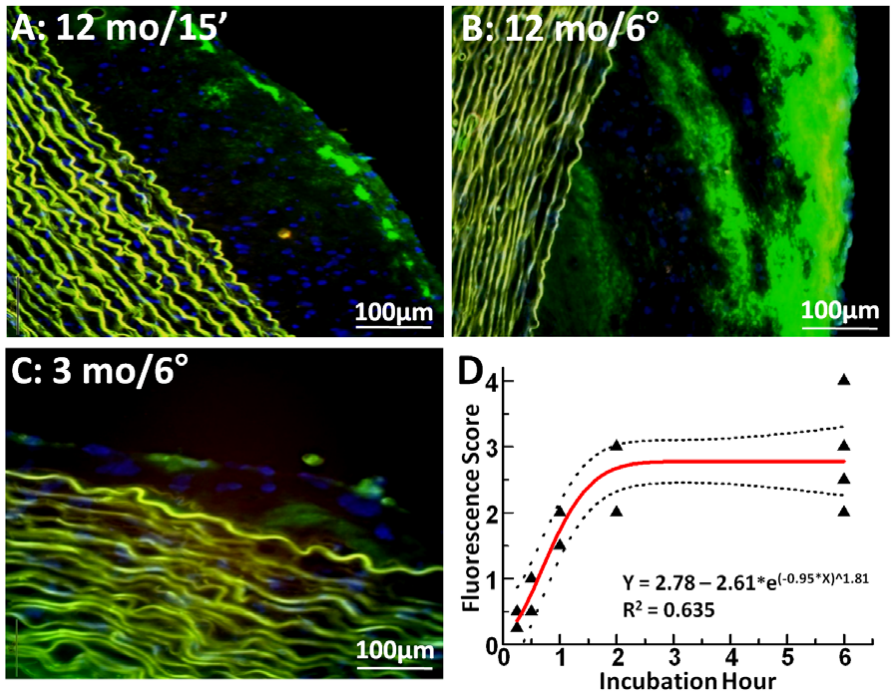
Fluorescence microscopy of rabbit aorta segments after *ex vivo* NP incubation for different times. A, B: 12 month cholesterol diet rabbit incubated with Alexa Fluor 488-labeled NP (green) for 15 minutes and 6 hours, showing progressively deeper penetration into intimal plaque region. Media shows elastin autofluorescence (yellow-green) only. Blue = nuclear stain C: 3 month cholesterol diet rabbit aorta incubated for 6 hours with Alexa Fluor 488-labeled NP showing minimal plaque thickening and only minor particle accumulation at endothelial surface. D: “Fluorescence score” of *ex vivo* NP-treated 12-month diet rabbit aorta segments incubated for different times, confirming greater penetration with longer NP exposure times. The exponential fit to the data indicates an asymptotic increase in penetration with little additional diffusion beyond 2 hour incubations.

To confirm that the nanoparticles entered the plaques rapidly by *passive diffusion*, *ex vivo* incubations of atherosclerotic aorta segments from two 9-month and five 12-month diet rabbits were carried out in tissue culture supported with *acellular* media. Aortic segments (n = 19) exposed to fluorescent nanoparticles for 15 min, 30 min, 1, 2 or 6 hours, revealed rapid penetration and trapping analogous to that observed for *in vivo* experiments (**Figure 4A, B**). Examination of 4 aortic segments from one 3-month diet rabbit undergoing *ex vivo* 2 and 6 hour nanoparticle incubations confirmed the minimal penetration previously observed for samples assessed after *in vivo* circulation (**Figure 4C**). MRI results again corroborated the fluorescence microscopy readouts of nanoparticle distributions (not shown). A plot of “fluorescence scores” for *ex vivo* incubated aortas at all time points (**Figure 4D**) illustrates the dependence of plaque permeability on the duration of *ex vivo* exposure to PFC-NP. ANOVA revealed a temporal dependence on fluorescence score (F = 8.0, p<0.002). No significant difference (p = 0.78) was observed between 2 hour and 6 hour *in vitro* treatment groups, consistent with the prior *in vivo* exposure data (see above), although the one hour incubations in this case were somewhat slower to particle diffusion (p = 0.06). These *ex vivo* incubations support the passive penetration hypothesis because there is no flow through vasa vasorum to transport the PFC-NP to the intima. Furthermore, the demonstrated gradient for PFC-NP accumulation is from lumen to intima, not from adventitia to intima.

### Structural features of disrupted endothelial barriers in rabbits

Histopathology: Adjacent sections were stained after fluorescent signal were confirmed. Basic plaque morphology is shown on H&E staining of a 12-month diet rabbit's aorta (**Figure S3A**). Traditional plaque constituents such as macrophages and foam cells were abundant (**Figure S3B**). Oil Red O staining demonstrated that lipid and cholesterol accumulations were distributed throughout the entire plaque (**Figure S3C**). Calcium staining localized near the media (**Figure S3D**). Smooth muscle actin was prominent in areas where nanoparticles were trapped after 24 hour *in vivo* circulation, as was glycosaminoglycan (**Figure S3E, F**), the presence of which in this model conforms with that described by Kolodgie et al in patient autopsy specimens [30]. However, none of these stainings suggested exact colocalization with the fluorescent nanoparticles, such that no clear assignment of a biophysical mechanism of trapping could be made based solely on histopathology. Tunel staining identified evidence of apoptotic cells in sections localized to the highly permeable plaque endothelium, with some evidence of cellular apoptosis within plaque intima (**Figure S3G**). Control rabbits were negative for apoptosis. PECAM-1 staining revealed prominent angiogenesis (**Figure S3H**) in the adventitia, but little in the intima until time points beyond 10 months (see below).

**Figure 5:**
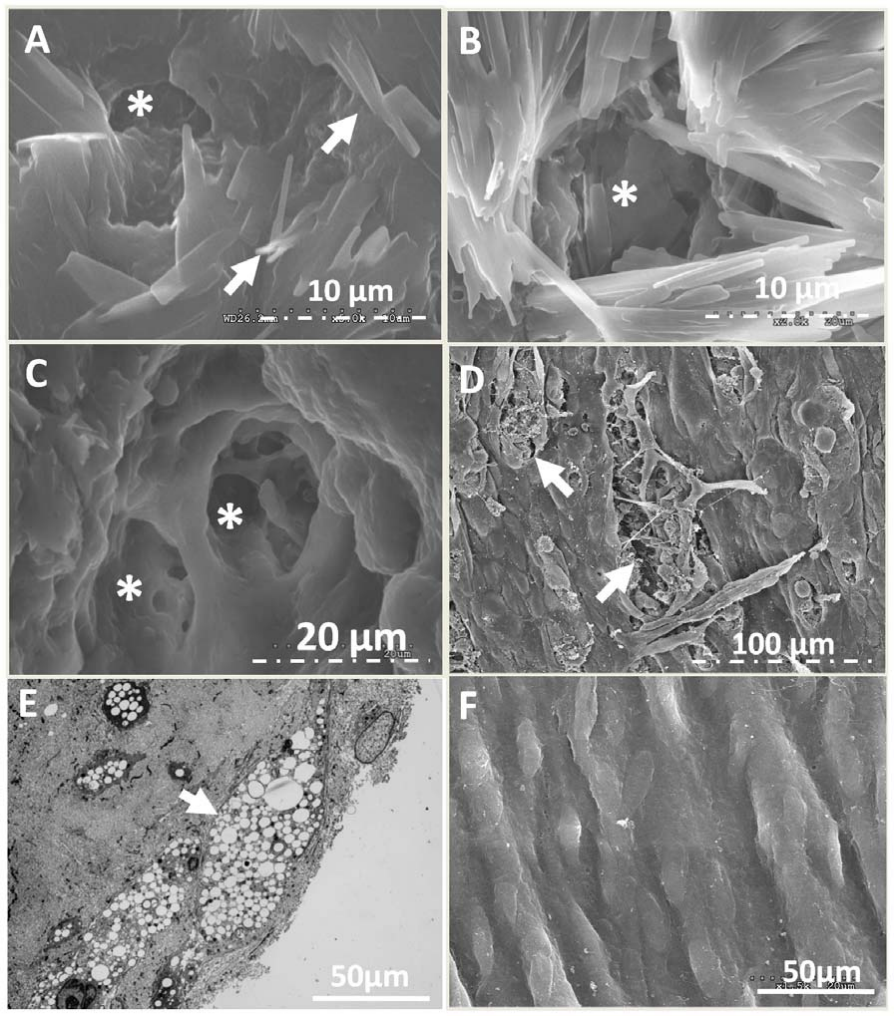
SEM images of rabbit aorta cholesterol plaque endothelium disruption. A: Speed vacuum dried, sputter coated SEM image of aortic endothelium of 12 month diet rabbit aorta demonstrating extensive superficial coating with crystalline structures (arrow) consistent with cholesterol that were not observed in standard ethanol dried preparations and similar to observations in human carotid endarterectomy samples made by Abela et al (see text). B: SEM images of speed vacuum dried cholesterol crystal around cavitary structure (*). C: Traditional dehydrated SEM image of cavitary structures (*) penetrating into subendothelial regions and surrounded by cholesterol crystals. D: Lower magnification SEM image with multiple endothelial erosions (arrow). E: TEM of endothelial foam cells (arrow) in cholesterol fed rabbit aorta sample. F: SEM image of normal aortic endothelium from regular diet rabbit for comparison.

Electron microscopy: Based on a recent provocative report by Abela et al of cholesterol crystals penetrating the endothelial surface of human plaques in unstable carotid artery disease [31], we sought to confirm the presence of these features of endothelial erosions in rabbits. Aortic specimens with obvious raised plaques were treated according to Abela's method to preserve the crystal structure by dehydrating tissues with speed vac drying, and some by the conventional ethanol dehydration method. Similar to what they observed in human tissues [31], cholesterol crystals penetrated the aortic endothelial surfaces of the rabbit plaques (**Figure 5A, B**). Intriguingly, we also observed numerous cavitary structures or channels literally, between and underneath the crystal structures. As Abela pointed out, SEM images on tissues processed by conventional ethanol dehydration destroys the crystal structures making them unapparent, but fortuitously enabling clear depiction of the superficial endothelial erosions (**Figure 5C, D**). The similarity of these structural features in rabbit plaques to human carotid artery samples derived from patients with acute vascular syndromes as reported by Abela is striking. TEM images of atherosclerotic vessels reveals the presence of endothelial foam cells (**Figure 5E**) that are associated with barrier dysfunction [32]. Although TEM-based identification of erosions can be challenging, we note that in sharp contrast to these superficial erosions observed in **Figure 5D**, control rabbit aortas exhibited smooth endothelial surfaces by SEM (**Figure 5F**) under all processing conditions, reducing the likelihood of tissue processing artifacts. These features also were not observed in adjacent segments of apparently normal or earlier stage plaques in the cholesterol fed rabbits. Taken together, these results suggest that endothelial barrier disruption in atherosclerotic rabbits is structurally similar to that in humans.

### Analogy to human vascular barrier disruption

**Figure 6:**
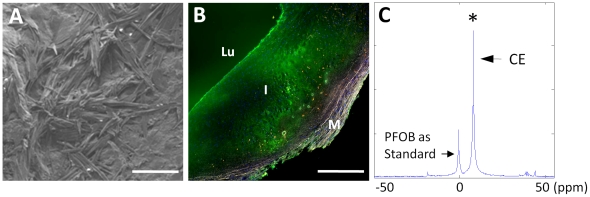
SEM, fluorescence microscopy and MRS of human carotid endarterectomy specimen. A: SEM image of intimal subendothelial cholesterol crystals in carotid endarterectomy tissue. (scale bar = 50 um) B: Fluorescence microscopy of human carotid endarterectomy specimen (fresh) incubated *ex vivo* with Alexa Fluor-488 labeled NP (green). Lu: lumen; I: intima; M: media. (scale bar = 500 um) C: ^19^F MR spectroscopy of human plaque segment indicating a strong signal and the presence of trapped CE NP in plaque. (ppm: part per million). PFOB standard signal from co-registered control sample is for NP calibration.

To relate these experimental findings to human atherosclerotic disease, carotid endarterectomy specimens from 8 patients undergoing clinically indicated vascular surgery were tested *ex vivo*. Freshly harvested segments for electron microscopy were fixed in 2.5% glutaraldehyde at 4°C for 24 hours and then speed vacuum dried overnight as outlined in the Methods section to permit observation of cholesterol crystals, which were similar to those observed in the rabbit tissues and in proximity to the disrupted endothelium (**Figure 6A**). Other fresh endarterectomy specimens were pretreated briefly with human plasmin (1 mg/ml) for 1 hour to digest residual superficial fibrin, and then incubated with fluorescent or non-fluorescent nanoparticles for 6 hours. Fluorescent microscopy indicated strong fluorescent signals from intact nanoparticles trapped in the intima (**Figure 6B**). Fluorine MRS confirmed that intact fluorescent nanoparticles penetrated into the plaques (**Figure 6C**). Specimens treated with non-fluorescent nanoparticles exhibited a strong ^19^F MRS signal, but no fluorescent signal (data not shown). Because these thin tissue sections for microscopy were cut from the center of the gross endarterectomy specimen by progressive slicing *after* incubation with PFC-NP, we note that the diffusion into the middle of the processed specimen represents true penetration into the mass of the plaque, which was devoid of adventitial microvessels.

### Nanoparticles penetrate passively from the lumen side through endothelial layer

*Ex vivo* control tests were performed to delineate the route of nanoparticle permeation. **Figure S4A** again shows the lack of autofluorescence in 12 month fed animal aortic segments. When the adventitia is manual stripped from the raised plaques in lesions from 2 aortic segments, leaving only the medial and intima to mimick the situation for the carotid endarterectomy specimens in patients , and then incubated for 6 hours prior to fixing and sectioning, a strong fluorescent signal is observed in the intima but not the media, suggesting that the nanoparticles readily can gain access from the luminal side, and not necessarily through vasa vasorum that might originate from the adventitia (**Figure S4B**).

To demonstrate that intimal permeation occurs by passive diffusion, sodium azide pretreatment of freshly excised plaques for one hour was employed to inhibit active cellular processes, which did not prevent nanoparticle penetration and trapping (**Figure S4C**). Furthermore, similar tests on formalin fixed aortic specimens demonstrated similar passive plaque penetration and trapping (data not shown). These tests indicate that active cell internalization and trapping is not required. Indeed, the PFC-NP appear to be distributed widely throughout all regions of the plaque in no direct association with any specific cell type, although some uptake cannot be ruled out but generally would not be expected after only an hour or so of *in vivo* circulation.

To further assess any potential relationship of nanoparticle permeation to plaque angiogenesis, PECAM-1 staining was performed in a random sampling of 24 aortic sections from 0, 3, 9 and 12 month fed rabbits (n = 3 rabbits each). Quantitative angiogenesis scores for 0, 3, 9, and 12 month fed rabbits were: intima: 0±0, 0±0, 0.67±1.2 and 3±1; and for adventitia: 5±0.7, 9.7±5.9, 7.2±4.6 and 4.3±1.5. Thus, the extent of neovasculature in the adventitia was considerable at all time points, but minimal in the intima until the later time periods. Because the NP penetration was maximal by 7 months at a time when intimal neovasculature was not well developed, and because NP penetration at 3 months was minimal at a time when adventitial neovasculature was maximized, NP permeation into the intima does not require intact blood circulation via plaque neovasculature. Taken together with the *in vitro* passive diffusion data in intact and adventitia-stripped rabbit aorta and in human specimens devoid of adventitia, it is reasonable to conclude that lumenal entry through disrupted endothelium predominates.

To define the severity of the barrier disruption in terms of size exclusion, we expanded the range of particle sizes and utilized selected dragon green-labeled polymer beads (53 nm and 2 um) on segments of 12 month diet rabbit aortas in 6 hour incubations (**Figure S5**). The smallest particles rendered a diffuse background staining extending to near the base of the plaque, while the larger 2 um particles were confined mostly to the more superficial aspects of the plaque. The PFC-NP penetration was intermediate to that of the smaller and larger structures, with some of the larger particles apparently distinguishable individually.

## Discussion

A principal physiological function of the vascular endothelium is to regulate trafficking of nutrients, fluids, signaling molecules, cells and such to and from the luminal space to the interstitial and cellular milieu of various organs and tissues. Yet, the mechanisms responsible for breakdown of this important barrier related to its timing, severity, and functional consequences remain poorly defined and virtually undetectable *in vivo* with any sensitive markers of endothelial disruption. Unfortunately, most atherothrombotic events occur without premonitory warning signs or symptoms and more than half of heart attack victims die suddenly without prior symptoms, involving an estimated prevalence of over 50 million such potentially “vulnerable” patients in America [33], [34]. In this report we have developed and demonstrated: 1) an *operational definition* of vascular barrier disruption based not simply on histopathology but on a quantitative diffusion-based methodology applied; 2) an animal model for endothelial barrier disruption that is progressive and appears to mimic aspects of human disease; and 3) a path toward an image-based method for evaluating endothelial barrier disruption that harbors clinical translational potential with the use of MRI-visible nanoparticles.

### Mechanistic implications of endothelial barrier disruption

The present report describes numerous structural and functional features of experimental endothelial barrier disruption that allows rapid penetration of nanoparticles, even up to micron size, far into the plaque intima both *in vivo* and *ex vivo*. These nanoparticles eventually are trapped and remain stably situated for at least 24 hours. Entry occurs by passive diffusion, and metabolic energy is not required, as shown by the *in vitro* incubations with azide pretreatment and in fixed tissues that show a similar appearance to the *in vivo* nanoparticle distributions. Thus, although *in vivo* convection might also facilitate entry, there is no requirement for cell based receptor-mediated binding of nanoparticles to permit plaque uptake.

The observed plaque surface erosions are associated with deep channel-like structures that could provide access to the plaque interior with dimensions that admit the larger sized (up to 2 micron) particles but not apparently red blood cells, since none were observed. The distribution of these erosions is heterogeneous and associated with cholesterol crystals penetrating the endothelium as has been reported for unstable human plaques [31]. In fact, the geometry is reminiscent of a spongiform complex, the surface area of which should be enormous relative to the surface area of the local plaque itself, offering significant potential for interaction with blood-borne molecular and formed elements of the clotting cascade. In contrast, relatively *intact* barrier function is observed in more normal appearing adjoining segments where nanoparticles are excluded from the intima, and severe plaque is not evident.

The state of endothelial disruption described in the current report might hypothetically represent an analogue of the advanced form of profound endothelial barrier disruption associated with the onset of plaque disruption in human disease. The temporal transition between minimal and severe barrier damage as detected by these types of nanoparticles occurs at some point between 3 and 7–8 months. The sigmoidal relationship in **Figure 2** suggests a critical period during which the endothelial barrier deteriorates and allows passage of these larger particulates, whereas an exponential fit might suggest a more gradual increase in permeability. Which scenario might be more applicable in patients is speculative.

At this disease stage, based on the present data, it should be advantageous to utilize larger nontargeted semipermeant nanoparticulates (e.g., 150–300 nm) that normally would not be expected to penetrate an intact or minimally disturbed endothelium to depict the severity of barrier disruption, and even to image it noninvasively with various molecular imaging approaches that are now being developed for clinical use [23], [24], [25], [35], [36]. Although much prior work has emerged for defining leaky vasculature in angiogenesis in cancer and cardiovascular disease with the use of highly permeable paramagnetic contrast agents that are used in standard MRI angiography, many feel that the appearance of these agents represent transport mostly across the plaque or tumor neovasculature [37], [38], [39], [40], [41], [42], [43]. However, if the endothelial barrier is disrupted it is possible that both avenues for contrast egress would exist and specific demonstration of luminal endothelial barrier disruption would not be possible with such small “extracellular” agents, but perhaps only with semipermeant larger nanostructures that also might permit quantification. In any event, the ability to quantify the signal based on ^19^F MRI/MRS could yield a solid metric for assessing the progression of disease with translatable imaging approaches.

It seems reasonable to infer that this model evolves in part as a rapid manifestation of cumulative cholesterol-related damage to the vascular endothelium, and thus barrier disruption might respond favorably to or be prevented by lipid lowering as suggested previously by others [44], [45], [46]. If so, the longer term feeding strategy employed here could represent a suitable experimental model of longstanding human disease demonstrable by its cholesterol crystal laden surfaces, endothelial apoptosis, and erosions. In focusing on the pathophysiological deterioration of the endothelium, new therapeutic targets emerge, such that if endothelial senescence could be mitigated as suggested by others [47], [48], disease progression might be slowed and the results monitored over time. At a minimum, such approaches may serve as useful tools for quantitative staging of the severity and progression of endothelial barrier disruption, and for defining the role of nanotechnologies for *in vivo* diagnostic imaging and therapy.

### Limitations

Although the short term NZW rabbit model of atherosclerosis is often considered as a type of storage disease rather than a model of complex human atherosclerosis, it is well accepted as a fundamental workhorse for experimental studies in numerous laboratories. Most of the data from rabbit models reported in the literature are acquired under conditions of shorter term (3–6 months) experiments, but these were not pursued as models of barrier disruption [49]. Ironically in our example, the longer term feeding and high cholesterol levels resulted in structural and functional analogues of human disease that created advanced endothelial barrier disruption. This strategy appears to compress the time frame over which severe endothelial damage emerges to less than a year (∼3–7 months), whereas in humans decades might be needed to produce the same extent of barrier disturbance.

Due to stringent preparation requirements for fluorescence imaging and accurate MRI/MRS quantification, we chose not to assess these distinct signals in the same aortic tissue segments. However, as both the fluorescence scores and the ^19^F signatures are unique with no obscuring background and thus definitive for the presence of PFC-NP, it is clear that in all aortic segments examined with either method that permeability in raised plaque regions was profoundly altered regardless of location, indicative of widespread barrier disruption as a function of long term cholesterol elevation. Although the development of robust *in vivo* imaging and spectroscopy was beyond the scope of the present investigation, these are subjects of future clinical investigation (see below).

### Clinical implications

The current technical advances point to several translational opportunities. The ^19^F MRI/MRS signatures are provocative because they are quantitative, exhibit no background single like ^1^H nuclei which are omnipresent, and manifest unique spectral signatures [36]. These types of particles have proven safe for systemic use in clinical trials as blood substitutes at >10× the present dose [50]. Clinical imaging solutions already exist for these and other types of particles [36], but assessment of their diagnostic utility for endothelial barrier disruption will require thorough testing *in vivo* in clinical trials. Indeed, such a trial ^19^F PFC-NP for integrin-targeted molecular imaging of carotid artery plaque with MRI/MRS has recently received IND approval by the FDA (#108,320). We propose that if this approach can identify and track the development of progressive vascular barrier dysfunction, it then could be tested for its predictive capacity and for following therapy designed to heal unstable or vulnerable plaques.

## Methods

All animal procedures were approved by the Washington University Animal Studies Committee (Protocol number: 2006 0145). For human endarterectomy tissues experiments, written consent was obtained from all patients undergoing clinically indicated surgical procedures, which included the use of excised deidentified tissue samples for research purposes. A protocol for the study entitled ‘Molecular Imaging of Cardiovascular Tissues’ was approved by Washington University Human Studies Institutional Review Board (HRPO# 09-0436).

### Animal model

Twenty-seven NZW rabbits were fed with a high cholesterol diet for 3–14 months (Modified Rabbit LabDiet® HF 5326 with 0.25% Cholesterol, Product #9344, TestDiet, Richmond IN). Serum total cholesterol levels were monitored periodically (Barnes-Jewish Hospital Clinical Pathology Laboratory). Control animals (n = 3) were fed with normal chow (**Table S1**). *In vivo* nanoparticle circulation was performed on 20 rabbits. Rabbit aortas were removed from the arch region beyond the left subclavian artery down to the level of the diaphragm (**Figure 1A**). Systematic evaluation of similar segments among all animals was performed from upper segments in order of sections for fluorescence microscopy and histology (1 cm), electron microscopy (0.5 cm), and lower segments for MRI/MRS and whole mount fluorescence imaging (IVIS) as shown in **Figure 1A**. Fluorescent *nontargeted* PFC-NP comprising CE cores developed originally for *in vivo* molecular imaging [24] (**Figure 1B** top left) were administered intravenously (1 ml/kg) and allowed to circulate for selected times ranging from 1–24 hours prior to aorta excision. The nominal particle diameter was 251.8±3.6 nm and polydispersity was 0.193±0.003 as measured by dynamic light scattering. **Figure 1B** bottom depicts the structure of the nanoparticles used. At selected time points, animals were euthanized and aortas were excised for further analysis.

Ten additional rabbits were used for *in vitro* confirmation of nanoparticle permeation of plaque (**Table S1**). Freshly excised rabbit aortic sections (n = 30, open lumen, 1 cm long) were incubated with fluorescently labeled PFC nanoparticles mixed with either rabbit blood or acellular media for 15 min to 6 hours to assess the time dependence of treatment. In some cases, aortic sections (n = 3) were first exposed to 5 ug/ml sodium azide [51] for 1 hour to inhibit active cellular processes. Tissue sections incubated in rabbit blood without nanoparticles or incubated in acellular medium with plain NP were used as negative control (n = 4). To examine a wider range of sizes for synthetic nanostructure permeability, commercially available fluorescent polymer particles of diameter 53 nm and 2 um (Bangs laboratories, Inc) were applied *in vitro* as above (n = 11 aortic segments from 3 rabbits with cholesterol diet for 12 months).

### Nanoparticle formulation

Previously described micro-emulsification methods were employed to formulate fluorescent PFC-NP of nominal diameter ∼250 nm [24], [25], [36]. No molecular targeting ligands were incorporated into the PFC-NP so as to permit assessment of passive diffusion and plaque retention. The fluorine-based particle cores comprised either CE or PFOB depending on use.

### Histology

Tissues for histological analysis were frozen in O. C.T media for fluorescence imaging. Cryosectioning (8 µm) was performed on 20 consecutive sections from each tissue sample, and 5–6 randomly selected sections were counterstained with DAPI for fluorescence microscopy and image digitization. Adjacent slides were saved for immunohistochemistry and special stainings.

Multiple images were acquired from each of the different sections with an Olympus BX61 fluorescence microscopy image system with Biological Suite software for microscope control and image processing (Olympus America, Inc, Center Valley, PA), using Color View II camera for optical image recording and digitization, and F-View II B&W CCD camera for fluorescent images. Images were merged from three channels of green, red, and blue, which localized nanoparticle signals, while also eliminating any autofluorescence, which was observed predominantly in the vessel media, and not in the intimal plaque region under these imaging conditions. Confocal microscopy was performed with an Olympus FV1000 microscope as noted. Whole mount fluorescence images were acquired with a Xenogen IVIS 100 system using Living Image 3.1 software.

The intensity and intimal penetration of nanoparticle fluorescent signals were analyzed using NIH Image J program and graded from 1–4, with each successive increment representing approximately 1/4 of the entire intimal plaque depth (or, from endothelium to internal elastic lamina) for a given plaque in a single section.

Thus, 1 = 25% plaque penetration, 2 = 50%, and so forth. No scoring was done for adventitia or for media because no significant nanoparticle fluorescent signal was associated with the signals in the same region of the plaque areas under any experimental condition. In specific, plaques that extended at least 20% around the lumen of the vessel were evaluated. Consensus between two experienced reviewers was achieved in all cases.

“Raised plaques” in this report are defined as a thickened intimal segment of artery that protrudes perceptibly from the normal smooth contour of the vessel extended outward into the lumen such that an obvious discontinuity exist at the shoulder region that departs from the otherwise smooth circumferential contour of the vessel intima (see **Figure 1C**). The additional presence of infiltrating cell nuclei stained blue (DAPI) in the intimal plaque regions (see **Figure 4**) confirms the presence of thickened intima in a raised plaque. Those areas of the arterial section directly adjacent or even remote that are not raised plaques were classified as grossly normal, although there may be some degree of more diffuse intimal thickening in these areas.

Stainings with oil Red O, Alcian Blue and Von Kossa were conducted following protocols of Laboratory Methods in Histotechnology (published by American Registry of Pathology, Washington, D.C.). Immunohistochemical staining for PECAM-1, Ram-11 (Dako, JC70A and MO633), and Alpha-SMA (Sigma, M-8421) were conducted using Vectastain ABC Kit (Vector Laboratory). An apoptosis detection kit (Chemicon, S7100) was used for TUNEL staining, especially for examining plaque endothelium in a limited sampling of aortas representing 0, 3, 9, and 12 months. For microvessel analysis, sections stained with PECAM-1 were quantified across the entire section for intima and adventitia separately, for a total of 24 sections and 3 rabbits from each diet period (0, 3, 9 and 12 month). Quantitative scoring was done by counting all stained vascular structures in each region of interest at 200× power.

Freshly harvested segments for electron microscopy were fixed in 2.5% glutaraldehyde at 4°C for 24 hours, and then half of each sample was speed vacuum dried overnight to permit cholesterol crystal detection according to the method of Abela et al. [31], the other half was dehydrated using the conventional ethanol preparation method. SEM images were acquired with Hitachi S-2600H and FEI Nova Nano 2300. TEM samples were prepared by core facility at biology department and images were acquired using Zeiss 902 electron microscope.

### MRI/MRS

*Ex vivo* ^19^F MRI/MRS of rabbit aorta specimens (n = 34 from 10 rabbits) and human endarterectomy tissues (n = 8 patients) were performed on an 11.7T Varian INOVA imaging console using a custom built single-turn solenoid coil (1 cm internal diameter). In each imaging experiment, ^1^H spin-echo images were first acquired to determine tissue anatomical appearance and location. Acquisition parameters of ^1^H MRI were: TR, 1 s; TE, 14 ms; slice thickness, 1 mm; field of view, 1×1 cm^2^; image matrix, 256×256; in-plane resolution, 40×40 µm^2^; and number of signal averages, 4. Subsequently, the resonance frequency of coil was tuned to 470 MHz for ^19^F spin-echo MRI at the same location. Acquisition parameters of ^19^F MRI were: TR, 1 s; TE, 14 ms; field of view, 1×1 cm^2^; slice thickness, 2 mm; in-plane resolution, 0.31×1.25 mm^2^ for cross-section view and 0.62×0.62 mm^2^ for projection view, and a scan time of 4.5 hours.

*Ex vivo* ^19^F MR spectroscopy was performed to quantify the ^19^F signal from tissue trapped nanoparticles with the following parameters: TR, 2 s; TE, 4 ms, 128 signal averages, and a scan time of approximately 4 minutes. Briefly, a ^19^F reference standard consisting 5 µL of 1% PFOB nanoparticle emulsion was placed with each tissue sample. Quantification of CE nanoparticles was based on the distinct ^19^F spectra of PFOB and CE according to the following equation^23^:
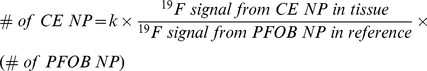
where *k* is a constant reflecting the ratio of ^19^F signal intensity acquired from the same amount of PFOB and CE nanoparticles. The number of CE nanoparticles in each specimen was normalized to the endothelial surface area. We note that previous studies comparing tissue concentrations of ^19^F (and PFC- NP) measured by spectroscopy and by gas chromatography of the PFC content have established the expected direct linear relation between the spectroscopic fluorine signatures and PFC-NP concentrations [27].

## Supporting Information

Figure S1 **Fluorescence microscopy and ^19^F MRI.** A: Intimal penetration of NP into aortic segment after 6 hours circulation in vivo (Alexa Fluor 488-labeled) in 9 month cholesterol diet rabbit aorta (green signal). Blue: DAPI nuclear stain, (scale bar = 100 um) B: Control study of *nonfluorescent* NP circulated for 6 hours in vivo in 9 month cholesterol diet rabbit aorta. Note lack of intimal fluorescent signal. Blue: DAPI nuclear stain. (scale bar = 100 um) C & D: Overlay of ^19^F (color) and ^1^H (gray) MR image (“en face” view) of the aorta tissues (same as A&B, respectively) confirmed strong heterogeneously distributed signals from trapped CE NP (color) in both samples. (scale bar = 3 mm).
(PDF)

Figure S2 **NP signals quantified by MRI/MRS.** A: Whole-mount surface fluorescence image over a grayscale coregistered photo of thoracoabdominal aorta from 10 month cholesterol fed rabbit after NP circulation in vivo for 1 hour (en face view). (scale bar = 10 mm) B: Local ^19^F (CE) MR spectroscopy of selected segment of aorta in C. An internal PFOB standard was used to enable quantification of CE NP shown in the chart. (scale bar = 2 mm) C: En face projection ^19^F MR image (color) overlaid onto ^1^H MR image (gray) of opened aortic segment (same as A) illustrates the heterogeneity of plaque and NP distribution. Various color bars in the right illustrate different possible metrics for NP quantification.
(PDF)

Figure S3 **Light microscopy for histological staining of aortic sections.** A: H&E staining of 12 month cholesterol diet rabbit aorta showing morphology of plaque intima. B: Ram11 staining of plaque macrophages and foam cells (brown). C: Oil red O stain of lipids (red). D: Van Kossa staining for calcification (black). E: Alpha-smooth muscle actin staining of smooth muscle cells (and myofibroblasts) (purple). F: Alcian blue staining for glycosaminoglycans (blue). G: Tunel staining manifesting endothelial cell apoptosis at plaque surface and intima (arrow). H: Pecam-1 staining with angiogenesis expressed on adventitia of 12 month cholesterol diet rabbit aorta (arrow). Lu: lumen; I: intima; M: media; A: adventitia.
(PDF)

Figure S4 **Control tests for NP penetration.** A: Ex vivo CE NP 6 hour incubation without fluorescent label in 12 month cholesterol diet rabbit aorta showing no intimal fluorescence signal, or autofluorescence (yellow), confirming that fluorescent signals originate from Alexa Fluor 488 labeled NP. B: Ex vivo 6 hour incubation with Alexa Fluor 488 labeled NP in 11month cholesterol diet rabbit aorta plaque stripped of adventitia, demonstrating equivalent NP penetration through the endothelium. C: Ex vivo 6 hour incubation with Alexa Fluor 488 labeled NP after 1 hour azide pre-treatment in 12 month cholesterol diet rabbit aorta does not affect the passive NP penetration and trapping. (scale bar = 100 um) Lu: Lumen.
(PDF)

Figure S5 **Fluorescent microscopy for a range of particle sizes penetrating plaque after 6 hour ex vivo incubation.** A: 53 nm diameter fluorescent polymer beads. B: 250 nm diameter CE nanoparticles labeled with Alexa Fluor 488. C: 2 um diameter fluorescent polymer beads. (scale bar = 100 um).
(PDF)

Table S1 **Experimental Subjects.**
(PDF)
