# Supplementary figures and images for: Quantifying the Evolution of Vascular Barrier Disruption in Advanced Atherosclerosis with Semipermeant Nanoparticle Contrast Agents
Source: PLoS One. 2011 Oct 18;6(10):e26385. doi: 10.1371/journal.pone.0026385 (PMC3196552; doi:10.1371/journal.pone.0026385)

Figure S1

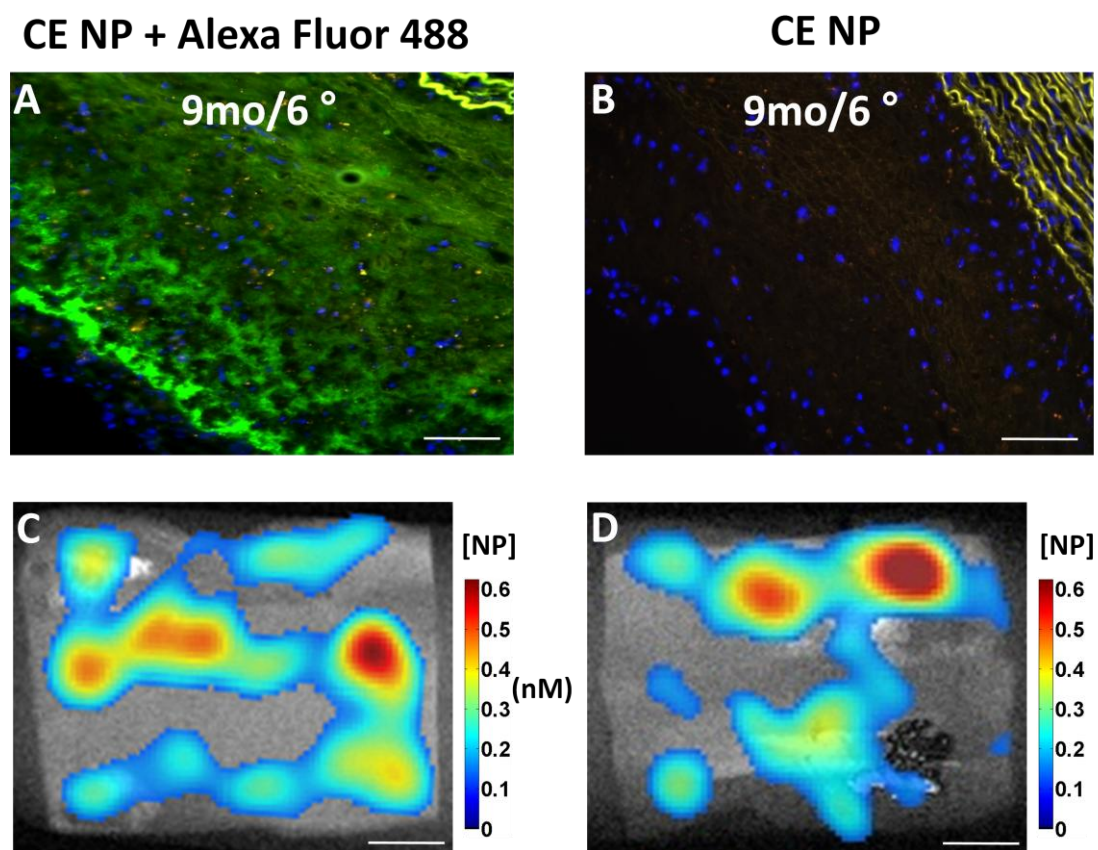

Supplement: Figure S1 — Fluorescence microscopy and 19F MRI. A: Intimal penetration of NP into aortic segment after 6 hours circulation in vivo (Alexa Fluor 488-labeled) in 9 month cholesterol diet rabbit aorta (green signal). Blue: DAPI nuclear stain, (scale bar = 100 um) B: Control study of nonfluorescent NP circulated for 6 hours in vivo in 9 month cholesterol diet rabbit aorta. Note lack of intimal fluorescent signal. Blue: DAPI nuclear stain. (scale bar = 100 um) C & D: Overlay of 19F (color) and 1H (gray) MR image (“en face” view) of the aorta tissues (same as A&B, respectively) confirmed strong heterogeneously distributed signals from trapped CE NP (color) in both samples. (scale bar = 3 mm). (PDF) [file pone.0026385.s001.pdf]

**Figure S2**

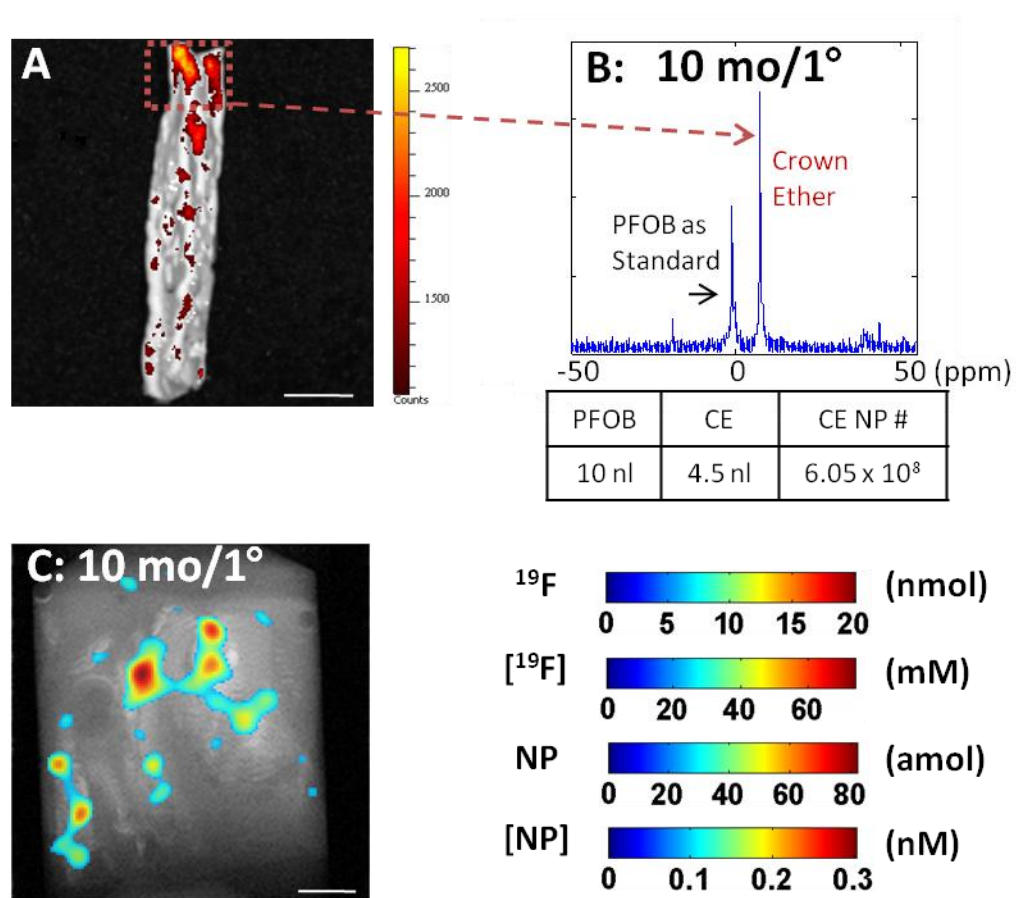

Supplement: Figure S2 — NP signals quantified by MRI/MRS. A: Whole-mount surface fluorescence image over a grayscale coregistered photo of thoracoabdominal aorta from 10 month cholesterol fed rabbit after NP circulation in vivo for 1 hour (en face view). (scale bar = 10 mm) B: Local 19F (CE) MR spectroscopy of selected segment of aorta in C. An internal PFOB standard was used to enable quantification of CE NP shown in the chart. (scale bar = 2 mm) C: En face projection 19F MR image (color) overlaid onto 1H MR image (gray) of opened aortic segment (same as A) illustrates the heterogeneity of plaque and NP distribution. Various color bars in the right illustrate different possible metrics for NP quantification. (PDF) [file pone.0026385.s002.pdf]

**Figure S3**

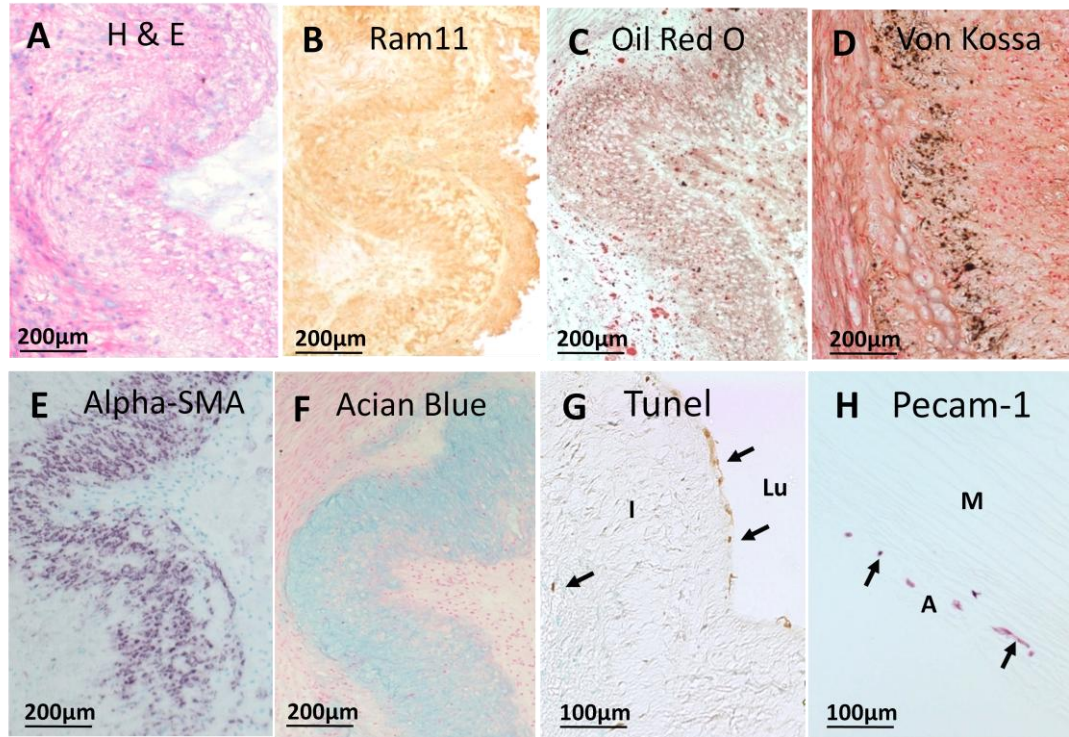

Supplement: Figure S3 — Light microscopy for histological staining of aortic sections. A: H&E staining of 12 month cholesterol diet rabbit aorta showing morphology of plaque intima. B: Ram11 staining of plaque macrophages and foam cells (brown). C: Oil red O stain of lipids (red). D: Van Kossa staining for calcification (black). E: Alpha-smooth muscle actin staining of smooth muscle cells (and myofibroblasts) (purple). F: Alcian blue staining for glycosaminoglycans (blue). G: Tunel staining manifesting endothelial cell apoptosis at plaque surface and intima (arrow). H: Pecam-1 staining with angiogenesis expressed on adventitia of 12 month cholesterol diet rabbit aorta (arrow). Lu: lumen; I: intima; M: media; A: adventitia. (PDF) [file pone.0026385.s003.pdf]

**Figure S4**

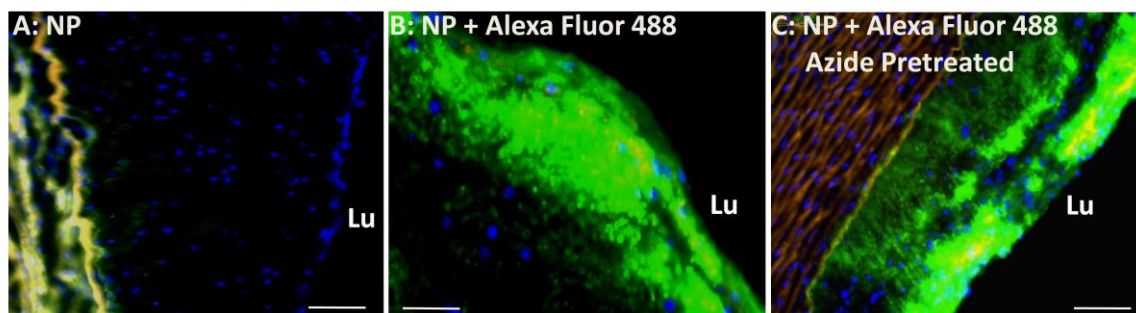

Supplement: Figure S4 — Control tests for NP penetration. A: Ex vivo CE NP 6 hour incubation without fluorescent label in 12 month cholesterol diet rabbit aorta showing no intimal fluorescence signal, or autofluorescence (yellow), confirming that fluorescent signals originate from Alexa Fluor 488 labeled NP. B: Ex vivo 6 hour incubation with Alexa Fluor 488 labeled NP in 11month cholesterol diet rabbit aorta plaque stripped of adventitia, demonstrating equivalent NP penetration through the endothelium. C: Ex vivo 6 hour incubation with Alexa Fluor 488 labeled NP after 1 hour azide pre-treatment in 12 month cholesterol diet rabbit aorta does not affect the passive NP penetration and trapping. (scale bar = 100 um) Lu: Lumen. (PDF) [file pone.0026385.s004.pdf]

**Figure S5**

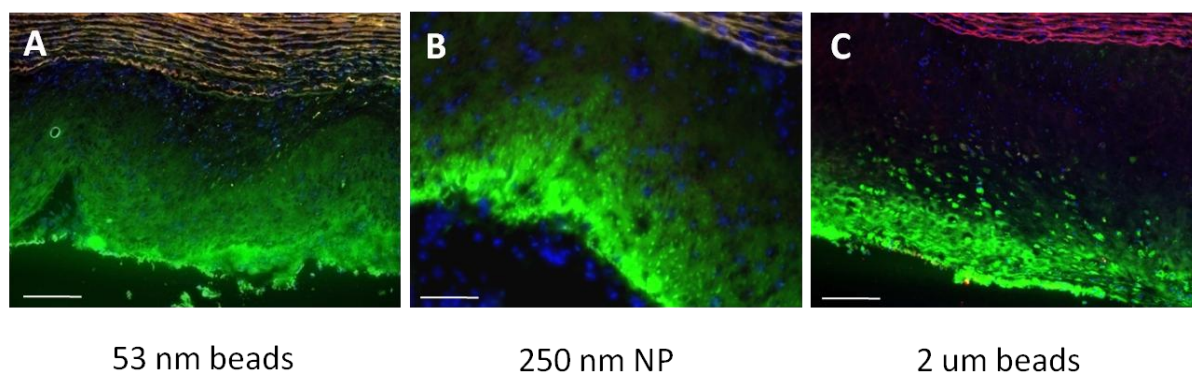

Supplement: Figure S5 — Fluorescent microscopy for a range of particle sizes penetrating plaque after 6 hour ex vivo incubation. A: 53 nm diameter fluorescent polymer beads. B: 250 nm diameter CE nanoparticles labeled with Alexa Fluor 488. C: 2 um diameter fluorescent polymer beads. (scale bar = 100 um). (PDF) [file pone.0026385.s005.pdf]
