# Supplementary material for: Quantifying the Evolution of Vascular Barrier Disruption in Advanced Atherosclerosis with Semipermeant Nanoparticle Contrast Agents
Source: PLoS One. 2011 Oct 18;6(10):e26385. doi: 10.1371/journal.pone.0026385 (PMC3196552; doi:10.1371/journal.pone.0026385)
Supplement: Table S1 — Experimental Subjects. (PDF) [file pone.0026385.s006.pdf]

**Table S1**

| In vivo | **diet** | **rabbits** |
|---|---|---|
| | 0M | 2 |
| | 3-5M | 7 |
| | 6-9M | 6 |
| | 12-14M | 6 |
| total | | 20 |

| Ex vivo | **diet** | **rabbits** | **sections** | **Fluor-NP** | **plain-NP** | **Beads** |
|---|---|---|---|---|---|---|
| | 0M | 1 | 4 | 4 | | |
| | 3-5M | 1 | 4 | 4 | | |
| | 6-9M | 2 | 12 | 11 | 1 | |
| | 12-14M | 6 | 25 | 11 | 3 | 11 |
| total | | 10 | 45 | 30 | 4 | 11 |
